# The Technical and Biological Reproducibility of Matrix-Assisted Laser Desorption Ionization-Time of Flight Mass Spectrometry (MALDI-TOF MS) Based Typing: Employment of Bioinformatics in a Multicenter Study

**DOI:** 10.1371/journal.pone.0164260

**Published:** 2016-10-31

**Authors:** Michael Oberle, Nadia Wohlwend, Daniel Jonas, Florian P. Maurer, Geraldine Jost, Sarah Tschudin-Sutter, Katleen Vranckx, Adrian Egli

**Affiliations:** 1 Clinical Microbiology, Cantonal Hospital of Aarau, Aarau, Switzerland; 2 Clinical Microbiology, labormedizinisches Zentrum Dr. Risch AG, Berne, Switzerland; 3 Institute for Environmental Health Medicine and Hospital Infection Control, University of Freiburg, Freiburg, Germany; 4 Institute of Medical Microbiology, University of Zurich, Zurich, Switzerland; 5 Clinical Microbiology, Dianalabs, Geneva, Switzerland; 6 Infectious Diseases and Hospital Epidemiology, University Hospital Basel, Basel, Switzerland; 7 Applied Maths NV, Sint-Martens-Latem, Belgium; 8 Clinical Microbiology, University Hospital Basel, Basel, Switzerland; 9 Applied Microbiology Research, University of Basel, Basel Switzerland; Swiss Institute of Bioinformatics, SWITZERLAND

## Abstract

**Background:**

The technical, biological, and inter-center reproducibility of matrix-assisted laser desorption ionization-time of flight mass spectrometry (MALDI TOF MS) typing data has not yet been explored. The aim of this study is to compare typing data from multiple centers employing bioinformatics using bacterial strains from two past outbreaks and non-related strains.

**Material/Methods:**

Participants received twelve extended spectrum betalactamase-producing *E*. *coli* isolates and followed the same standard operating procedure (SOP) including a full-protein extraction protocol. All laboratories provided visually read spectra via flexAnalysis (Bruker, Germany). Raw data from each laboratory allowed calculating the technical and biological reproducibility between centers using BioNumerics (Applied Maths NV, Belgium).

**Results:**

Technical and biological reproducibility ranged between 96.8–99.4% and 47.6–94.4%, respectively. The inter-center reproducibility showed a comparable clustering among identical isolates. Principal component analysis indicated a higher tendency to cluster within the same center. Therefore, we used a discriminant analysis, which completely separated the clusters. Next, we defined a reference center and performed a statistical analysis to identify specific peaks to identify the outbreak clusters. Finally, we used a classifier algorithm and a linear support vector machine on the determined peaks as classifier. A validation showed that within the set of the reference center, the identification of the cluster was 100% correct with a large contrast between the score with the correct cluster and the next best scoring cluster.

**Conclusions:**

Based on the sufficient technical and biological reproducibility of MALDI-TOF MS based spectra, detection of specific clusters is possible from spectra obtained from different centers. However, we believe that a shared SOP and a bioinformatics approach are required to make the analysis robust and reliable.

## Introduction

Matrix-assisted laser desorption/ionization-time of flight mass-spectrometry (MALDI-TOF MS) is commonly used for the rapid identification of bacterial species via characterization of highly specific protein mass-spectra profiles[1,2]. For microbiological routine diagnostics, the MALDI-TOF MS technology focuses on a mass-range between 2 to 20kDa, covering mainly ribosomal proteins harbouring a high diversity between species[3–5]. In addition, this technology can also be used for typing purposes at a sub-species level. Most publications focused on an improved resolution of species identification within a bacterial complex or group e.g. *Bacillus cereus* group[6,7], *Burkholderia cepacia* complex[8,9], or Mycobacterium *abscessus* complex[10,11]. In addition, several reports have indicated that MALDI-TOF MS based typing may be used for rapid infection control investigations including a wide range of bacterial species[12–20]. The technology allows the comparison of mass-spectrometry spectra data with a potential resolution down to a single amino acid difference between outbreak strains and non-related isolates. The range and frequency of shifts in the mass-spectra peak profiles can be used to determine the relatedness between single isolates within an outbreak. We have recently established a standard operating procedure to type extended spectrum β-lactamse (ESBL)-producing *Escherichia coli* and could show a highly similar dendrogram compared to pulsed field gel electrophoresis[15].

However, in none of these studies the overall technical, biological, and inter-center reproducibility of a same set of bacteria has been addressed. This would be a crucial step to validate the robustness of MALDI-TOF MS based typing. An investigation in its robustness would allow further implementation of this new and rapid typing method. It would also allow establishing a protocol to prepare the samples for sub-typing. In addition, interpretation of complex MALDI-TOF spectra in the context of typing application may be challenging [21].

We hypothesize, that (i) the variance at a sub-species level may be determined by using MALDI-TOF MS, and (ii) the results are dependent on high quality profiles including a technical and biological reproducibility, followed by a bioinformatics work-up. Therefore, we aimed to assess the technical, biological and inter-center reproducibility of MALDI-TOF MS based typing in a blinded multicenter study including six microbiology diagnostic laboratories examining two nosocomial outbreaks and two non-outbreak related isolates of ESBL-producing *E*. *coli*.

## Materials and Methods

Bacterial isolates. We included twelve isolates of ESBL-producing *E*. *coli* collected of two hospital-related outbreak clusters (six and four each) and two non-outbreak related isolates from the University Hospital Basel and the Cantonal Hospital Aarau. The non-outbreak related isoaltes were from urinary tract isolates of the University Hospital Basel. The relatedness of the isolates was previously analysed with pulsed field gel electrophoresis (**S1 Fig**). These isolates were sent to the six participating diagnostic microbiology laboratories (see affiliations). The centers were blinded for the outbreak and non-outbreak related isolates. In one center, two technicians independently performed MALDI-TOF MS typing, resulting in two data sets from the same center (referred to as center 5A and 5B). All participating centers used the Microflex MALDI-TOF MS system (Bruker Daltonics, Bremen, Germany). Technicians had low or no experience in MALDI-TOF MS based typing. Therefore, we used a previously published standard operating procedure (SOP) to provide a protocol guiding, step by step, through the whole procedure [15]. All participants were blinded regarding the relatedness of the isolates. All peak profile raw data from each center can be downloaded here: https://figshare.com/articles/AllRawSpectraEgliPlosOne_zip/3749454

SOP. Each center received a SOP for the exact handly of the samples. The SOP was explained by a common instructor. Briefly, all bacterial isolates were stored at -80°C, thawed and sub-cultivated prior to sending to the participating laboratories. Each participating laboratory re-cultivated the isolates at standard conditions on a blood agar plate in an aerobic atmosphere at 37°C for 18–20h. All twelve isolates had to be sub-cultivated simultaneously to ensure the same age among the colonies to control the senescence-associated changes in the mass peak spectrum. Twenty ul of bacterial colony material (corresponds to a full 1μL loop) was used for ethanol-formic acid (70%) protein extraction [15]. For each protein extraction, four separate spectra were recorded (quadruplicates) using the FlexControl software (Bruker Daltonics, Bremen, Germany) to assess technical reproducibility. The whole analysis (cultivation, extraction, and spectrum generation) was repeated between two and five times independently at each laboratory to assess the biological reproducibility. Species had to be confirmed in comparison with the mass-spectrum library using the MALDI Biotyper 3 software (OC 3.1, Bruker Daltonics) at standard conditions.

Visual analysis. Each participant visually read the spectra of the 12 isolates in the flexAnalysis software (Bruker) in the overlay mode to establish a peak list. Each single isolate and each measurement was inspected individually. Peaks below 2500 m/z were ignored for the analysis. Peaks with intensities above 1000 arbitrary units were included for the analysis. Masses, where peaks were present in some of the isolates but absent in other isolates were defined as discriminant peaks which were noted in an Excel sheet. The final table was sent for analysis and comparison. For all center the peaks with the highest discriminatory power was determined and the peaks were compared.

Bioinformatic analysis. In contrast to the participant’s own interpretation and allocation of peaks for each isolate (subjective interpretation), a more detailed bioinformatics approach provided an objective interpretation of the raw data. Each center provided the recorded peak profiles (raw data file from Bruker) and these were analysed first to confirm the bacterial species using the library database. This analysis was performed with the flexAnalysis Software (v3.4, Bruker). For further bioinformatics the BioNumerics software (Applied Maths, v7) was used allowing a detailed analysis of the complex mass spectra profiles including thousands of peaks. Before analysis the profiles were smoothed and baseline peak shifts were subtracted, which is a software feature either in the Bruker flexAnalysis software or in the BioNumerics Software.

The technical reproducibility was measured calculating the average similarity of each spectrum to the other technical replicates using the Pearson correlation coefficient. The average similarity was the comparison of each spectrum of a quadruplicate from one individual center summarized to an average spectrum, which can be generated with the BioNumerics Software. The spectra are only included if they meet the filtering criteria for minimum intensity and minimum similarity to the summary spectrum of all technical replicates. The minimum intensity was arbitrarily set to a peak intensity of 1000 units. The similarity of a particular peak in comparing spectra had to be in a 1–5 Dalton range; peaks within a higher rang were identified as separate peaks. The filtering criteria influenced directly the specificity for a particular peak and spectrum. The reproducibility on biological level was measured by first calculating a summary spectrum based on all included technical replicates, followed by comparing the summary spectra of biological replicates to each other. The average similarity of the biological replicates was calculated with the Pearson correlation coefficient.

Peak matching: Kruskal-Wallis was used to determine significant difference between spectra of different clusters. Data was normalised to the average intensity per spectrum and log transformed (base 2). The resulting data followed a normal Gaussian distribution (checked with the Kolmogorov test) and an Anova was performed to detect peaks that were significantly different between the clusters. The peaks obtained from both Anova and Kruskal-Wallis were combined. This resulted in the identification of discriminating peaks. These peaks were further analysed using the TagIdent online tool (http://web.expasy.org/tagident/) for bacterial protein allocation.

Support Vector Machine: To determine the ability to discriminate the different outbreaks, the dataset of center 5 was considered as the reference data and used to train a linear Support Vector Machine classifying algorithm (SVM). After training, an internal validation was done by removing each spectrum individually, treating it as unknown and classifying it with the trained SVM. For each entry, the score with its own group and with the best scoring other group was determined using the p-value (chance that the entry belongs to that group) as calculated by the SVM. For a good discrimination, the score with its own cluster should not only be highest, but there should be a large contrast between both scores. An external validation was done by classifying all the spectra from the other centers using the trained SVM and comparing the groups predicted by the SVM with the actual group.

## Results

### Technical and biological reproducibility of MALDI-TOF MS typing data

**Fig 1:**
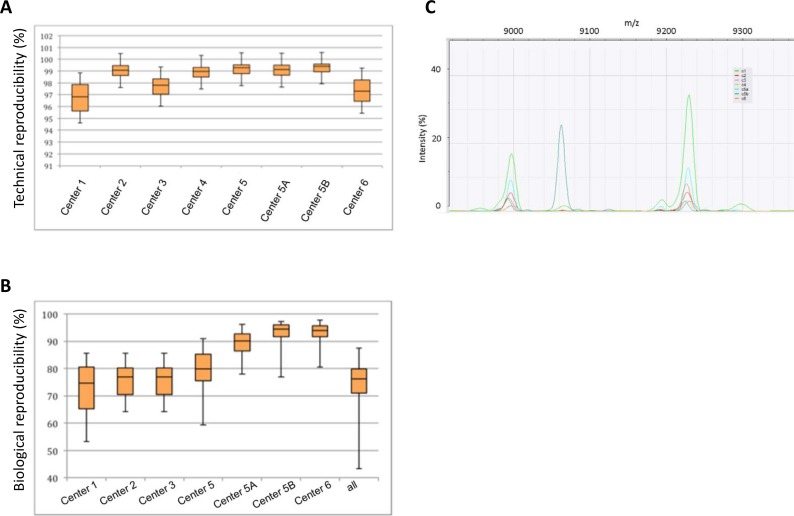
Reproducibility of MALDI-TOF MS based typing data. Box-plots show the average similarities in percentage. Median and interquartile range is indicated, whiskers indicate the range. Numbers indicate the different centers, (e.g. center 1 etc.); center 5A and 5B indicates that two microbiologists independently performed the experiment at center 5. **(A)** Technical reproducibility in percent. **(B)** Biological reproducibility in percent. **(C)** Peak comparison between centers. Three peaks are shown: peaks at 9000 and 9220 m/z are detected by all centers, however the peak at 9060 is only detected by one center.

Fig 1A provides an overview of the technical reproducibility (**Fig 1A**). The number of biological replicates varied slightly between centers (range 2 to 5 times). Fig 1B provides an overview of biological reproducibility (**Fig 1B**). Interestingly, the data from center 5 showed the effect of two technicians running the samples. Both technicians have very high technical and biological reproducibility, but pooled together, the biological reproducibility shows a drop. Some peaks were only detected in individual centers, whereas most peaks were detected by most centers–an example is shown in **Fig 1C**.

### Subjective interpretation by visual analysis

Each participant visually interpreted the spectra of the 12 isolates and established a peak list. The summary of the peak lists from all centers resulted in 22 identified peaks. Five of these 22 peaks were noted once as discriminative by centers as with a 14% rate of agreement (**S1 Table**). In contrast, six of 22 peaks were identified by centers as clearly discriminant masses with a high rate of agreement. Three peaks among them (m/z 6539, 8350, and 9712) were 100% concordant between all centers, representing peaks with high impact for strain-discrimination. The subjective interpretation of larger datasets by visual analysis is challenging for un-experienced or blinded participants. Therefore, we employed bioinformatics analysis on the raw data collected from each center to enrol an objective interpretation. This included based on the technical and biological replicates between 48 and 235 spectral profiles per center resulting in a total of 1044 analysed spectra. In total, 22’968 single mass peaks were analysed and compared.

### Reproducibility of MALDI-TOF MS typing data between different centers

The Pearson correlation coefficient clustered isolates 5–10 together in all centers (outbreak cluster 2). For most centers, isolates 11 and 12 (non-outbreak related isolates) also clustered together, as well as samples 1–4 (outbreak cluster 1). The cluster 1 and the isolates 11 and 12 were more closely related to each other than to the cluster 2 (isolates 5–10). Exceptions to this clustering are shown in the dendrograms of each center as supplementary material (**S2 Fig**).

A clustering of all spectra from all centers did not contain the different sub-clusters mentioned previously (**S3 Fig**). Most spectra showed a tendency to cluster together among spectra from the same center.

**Fig 2:**
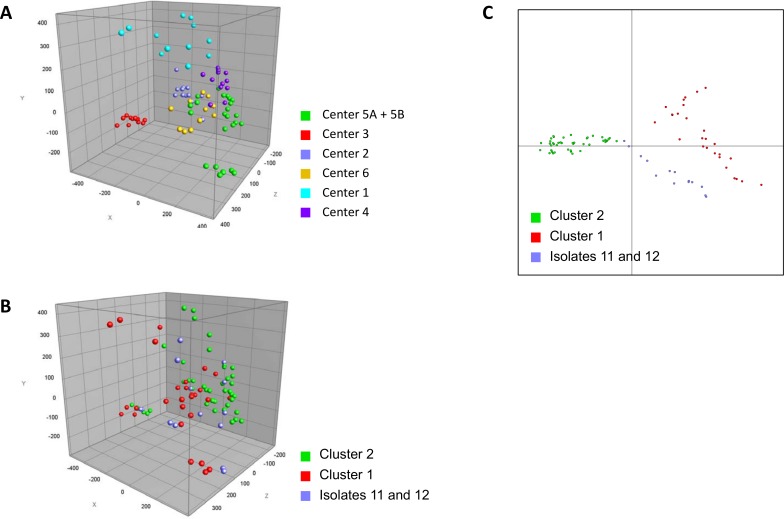
Principal component and discriminant analysis. **(A)** Principal component analysis (PCA) indicates a strong center specific effect. Each center is depicted with a different colour code, e.g. red data points clusters together and do not allow to separate clusters independent of each center. (**B**) PCA within the groups of all clusters implicates some difference, however the clusters cannot reliably be separated. (**C**) The discriminant analysis highlights that the clusters can be separated. Each cluster is shown with a different colour code.

This was also confirmed with a principal component analysis (PCA), which showed that indeed spectra had a higher tendency to cluster from the same center (**Fig 2A and 2B**). This implies that the technical variation between centers is too high to simply compare complete spectra. However, performing a discriminant analysis on the complete dataset did show a comprehensive separation of the two outbreak clusters and the isolates 11 and 12 (**Fig 2C**). This indicates that the separation of the two outbreak clusters based on the spectra from the different centers is possible.

### Identification of cluster separating peaks

The next approach was to identify peaks responsible for separating the clusters. First, we determined center 5, where two technicians analysed the spectra separately, as reference center. These data sets were used to detect the clusters and to define specific peaks to alocate spectra coming from different centers to the correct clusters. A peak matching was performed on all the spectra from the center 5 with a constant tolerance of 2 m/z and a linear tolerance of 500 ppm. This analysis resulted in the identification of totally 12 peaks. Each peak was visually inspected on the spectra to confirm the difference was not the result of minor intensity variation within the expected technical variation. All peaks were withheld based on this inspection.

**Fig 3:**
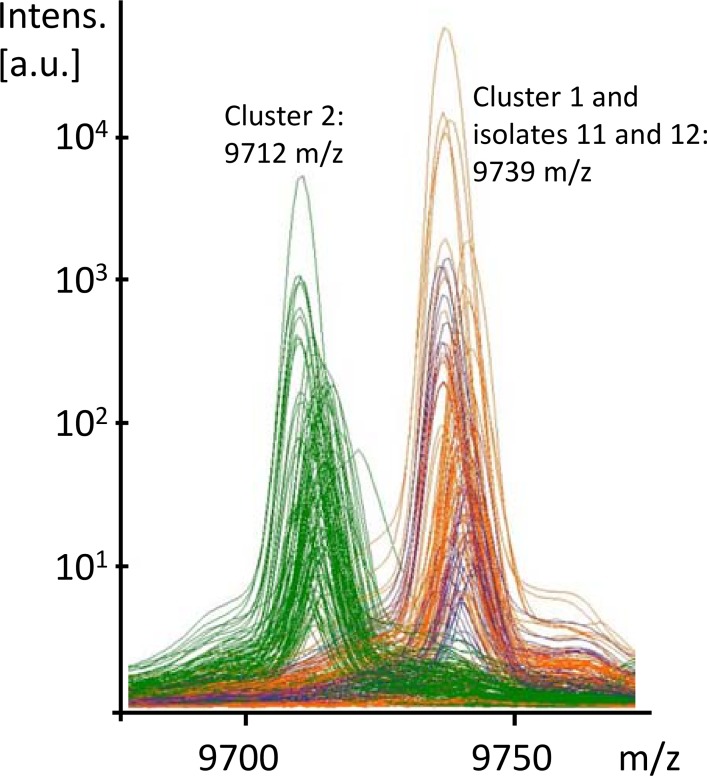
Peak comparison. Identification of cluster-separating peaks. The x-axis indicates the m/z value and the y-axis the peak intensity. Alternative 1: Single line analysis shows an extract of three peak profiles with a ‘peak pair’ at 9712 and 9739 m/z. The peak on the left part of the graph shows a shift in the top two spectra compared to the bottom spectrum, while the peak on the right is in the same position for all three. Alternative 2: The overlay mode of multiple spectra show ‘peak pairs’ at 9712 and 9739 m/z. Each line reflects a single measurement. The green lines are spectra of cluster 2-isolates.

In this set of peaks, several ‘peak pairs’ were observed. We determined two peaks a peak pair if they were very close to each other, typically within a 20–50 Dalton range and when their presence and absence was inverted between datasets. This is usually the result of nucleotide polymorphism in the bacterial gene, causing an amino acid change in the protein. For example, CAC is mutated to AAC, which results in Histidine to Asparagine change and thereby also a shift of the size of the protein (minus 23 dalton). Fig 3 shows close up of such a peak pair (9712 and 9739 m/z) (**Fig 3**). For some peaks, a matching peak was observed in the spectra, but the other peak did not reach a significant level in terms of intensity. This was mainly the case if a peak was very close to the detection limit and was not detected in all samples. In these cases the matching peak was still considered specific for a cluster and included in the final set of peaks. This approach resulted in the identification of two additional peaks. The set of 12 peaks was then matched to the complete set of spectra from all centers. Table 1 shows a summary of all peaks and allocations to the two clusters and the isolates 11 and 12 (**Table 1**).

**Table 1:** Peaks identified to separate the clusters.

Peak Position	Cluster 1	isolates 11 and 12	Cluster 2	Possible proteins (from TagIdent)
3444	Yes	Yes	No	Protamine-like protein
5873	Yes	Yes	No	Regulatory protein MokB
6539	Yes	No	No	50S ribosomal protein L30
7173	Yes	No	No	Pilin; Protein CopA/IncA
7650	No	No	Yes	Response regulator inhibitor for tor operon; Protein KleB; Protein IscX; Cold shock-like protein CspH
7708	Yes	Yes	No	Response regulator inhibitor for tor operon; Protein KleB; Protein IscX; Cold shock-like protein CspH
8326	Yes	Yes	No	Tautomerase PptA; Dihydrofolate reductase type 2; Ferrous iron transport protein A
8350	No	No	yes	Tautomerase PptA; Dihydrofolate reductase type 2; Ferrous iron transport protein A
9712	No	No	yes	30S ribosomal protein S17; Regulatory protein AriR; UPF0386 protein YjhX; Acid stress chaperone HdeA
9739	Yes	Yes	No	30S ribosomal protein S; Regulatory protein AriR 17; UPF0386 protein YjhX; Acid stress chaperone HdeA
10463	Yes	Yes	No	30S ribosomal protein S19; Sugar fermentation stimulation protein B
10489	No	No	Yes	30S ribosomal protein S19; Sugar fermentation stimulation protein B

S3 Fig shows the peak matching results and clustering based on this peak matching (**S4 Fig**). Cluster 2 could be clearly distinguished in this dendrogram. The only outlier was sample 7 from center 2, which has a very low overall intensity, with too few peaks being above the detection limit.

### Classifier algorithm to separate the peak spectra of closely related clusters

**Fig 4:**
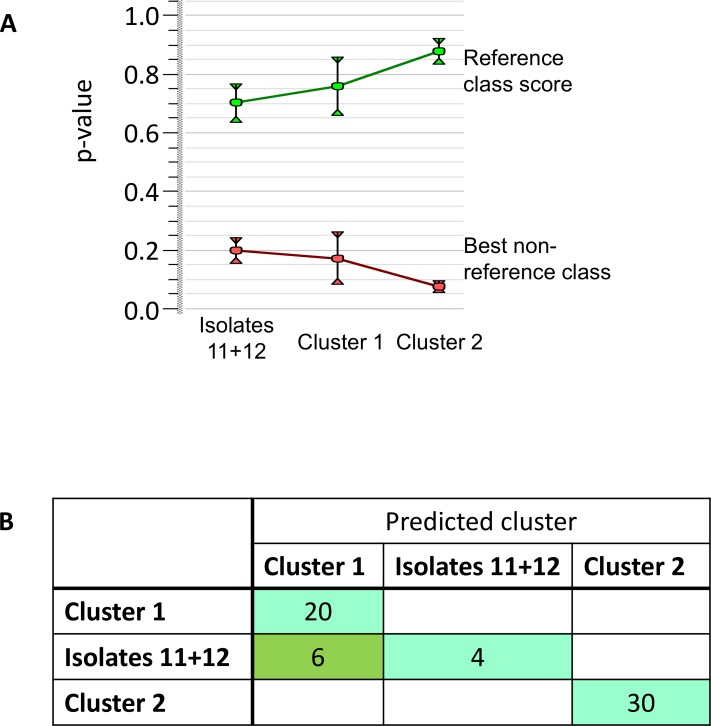
Classifier data analysis. (A) Separating capacity for reference center: average scores (p-values) and standard deviation with the own group of the spectra (green) and with the best scoring other group (red). For each group the score with the own group is highest with no overlap in the standard deviation of both scores. (B) Separating capacity for all centers: the number of isolates belonging to a certain predicted group by SVM per group they actually belong to. The diagonal contains the number of correct identifications per group, outside the diagonal are the incorrect identifications.

Classifiers algorithms are very good at distinguishing between groups even though the differences are minimal. A support vector machine is specifically useful for this type of data [22]. Therefore, an identification project was constructed using the spectra from center 5 as reference set and a linear support vector machine as classifier. An internal validation showed that within the set of center 5, the identification of the cluster was 100% correct with a large contrast between the score with the correct cluster and the next best scoring cluster (**Fig 4A**). As expected, the score contract was highest for outbreak cluster 2. This identification project was then applied to all spectra from different centers. As expected, cluster 2 was identified correctly for all sets from all centers. However, cluster 3 was misidentified as cluster 1 for three centers (center 1, 3, and 6) (**Fig 4B**). These centers were also the ones with the lowest technical reproducibility (compare **Fig 1A**).

An identification project containing the spectra for all centers further reduced the number of misidentifications, to just two isolates among the two non-outbreak related isolates based on the internal validation.

## Discussion

Our MALDI-TOF MS based typing approach showed that the technical and biological reproducibility between different centers is sufficient to allow the detection of distinguished clusters. In general, the clustering obtained using a Pearson correlation coefficient on the summary of biological replicates in each center was comparable, though some exceptions occurred. Even though the individual clustering in each center was comparable, the comparison of data between different centers could not be done in this way. In particular, the detection of outbreaks by comparing the complete spectra using the Pearson correlation coefficient was considered unsuitable, if the data comes from different centers.

Some bioinformatics techniques are required to make the analysis robust and reliable. Visual inspection of large datasets is time consuming and often not possible. In our dataset almost 23’000 single spectra peaks were included. The more closely related different clusters are to each other, the less reliable their separation is. The role of the technical reproducibility seems to be the most critical part and should be further investigated. In particular, future studies should focus on determining whether increasing the technical reproducibility also leads to a more reliable separation of clusters and which steps in the preparation of the samples and detection of the spectra have the most influence on the reproducibility.

Even after peak matching, clusters 1 and the two non-outbreak related isolates were still grouped closely together. This was not very surprising as the difference was only two peaks that were present in cluster 1 and absent in the two non-outbreak related isolates. For one of these peaks, the presence in cluster 1 was not consistent. In a clustering analysis, these minimal differences were overruled but the technical variation between the peak detection in the difference spectra. Therefore, we needed a more sensitive technique to distinguish between these groups. Finally, with a classifier algorithm the difference between clusters could be reliably determined.

Using a SOP may pose an important step for successful MALDI-TOF MS based typing. In particular the same age of bacterial subcultures, a “full protein extraction” protocol, and inclusion of non-related isolates is crucial in the typing process. Reproducibility of typing data will be a key element in establishing this potentially new typing method in the future. However, the SOP alone did not provide sufficient impact for each individual center. Veenemans and colleagues have compared different growth conditions and culture media for ESBL-producing *E*. *coli* and showed substantial differences [23]. In the future it will be important to further standardize the protocols.

For typing, isolates representing outbreak strains would ideally be indistinguishable from each other and highly diverse from those of non-related strains. For PFGE-based typing, significant differences have been defined as distinctions in at least seven bands [24], corresponding to an approximately 80% similarity between isolates. Whole genome sequencing shows the highest resolution for typing and even single nucleotide polymorphisms not affecting the amino acid sequence can be depicted and analysed. However, this method is not yet available for many laboratories and still expensive. MALDI-TOF may have a role in quickly assessing potential groups, which then can subsequently be analysed with higher resolution typing methods. For MALDI-TOF MS based typing the sensitivity of differentiation is highly dependent on the peak spectra quality. One peak shift might indeed provide evidence that two isolates are different. Using classifier algorithms and high quality data (with high technical reproducibility >98%) allows a clear determination of outbreak peaks–in this case using the BioNumerics software allows a calculation of similarity with a Pearson correlation method.

Our study has a number of limitations. First, this was only a set of data focusing on one pathogen, which may be more conducive to taking a MALDI-TOF MS based approach. An important limitation is the small sample size included into the study and therefore this may indeed limit generalizability of our results to other settings. However, with a high technical reproducibility different centers could clearly allocate individual strains into the correct clusters. In addition, the generated data-points of each single protein profile in technical quadruplicates and biological triplicates generated a significant dataset, resulting in almost 23’000 single peaks to analyse, which individually can serve as a biomarker to address the various aspects of reproducibility. Nevertheless, it will be very important to provide further studies comparing much larger sample sizes and different technical protocols are needed [25]. Peak shifts only occur in missense mutations with changes of the amino acid sequence, therefore in comparison to genetic based typing silent mutations will be missed [26]. However, we strongly believe that these methods might be used complimentary–especially when genome based typing is not available, MALDI-TOF MS based typing might be able to rapidly assess a potential outbreak. The bioinformatician was aware about the clustering of the isolates, therefore this might have introduced a potential bias in choosing particular bioinformatics tests.

Overall, our multicenter validation study highlights that the identification of similarities and differences between ESBL *E*. *coli* strains is possible using MALDI-TOF MS. This novel approach to outbreak investigation may allow real-time typing, therefore revolutionizing outbreak investigations. In contrast to next generation sequencing based technologies, this approach is significantly cheaper and faster. In a potential outbreak situation MALDI-TOF MS based typing might provide first evidence to then initiate a more complex and higher-resolution typing technology.

## Supporting Information

S1 Fig Pulse field gel electrophoreses (PFGE) of the isolates. Two outbreak clusters and two non-outbreak related isolates were included into the PFGE analysis. The isolates of the outbreak clusters nicely cluster together (>95% similarity in a pearson’s analysis). The outbreaks were also epidemiologically linked (data not shown).
(TIFF)

S2 Fig Dendrogram analysis of center 1–5 for the two outbreaks and non-outbreak related isolates. A-G show individuals centers. **A**, center 1; **B**, center 2; **C**, center 3; **D**, center 4; **E**, center 5A; **F**, center 5B.
(TIFF)

S3 Fig **Dendrogram analysis of center 6 and overview A**, center 6. **B** shows the overall dendrogram including all data from all centers.
(TIFF)

S4 Fig Peak matching results and respective clustering. (TIFF)

S1 Table Summary of subjective interpretation of discriminant peaks by each center. (DOCX)
