# Supplementary figures and images for: The Technical and Biological Reproducibility of Matrix-Assisted Laser Desorption Ionization-Time of Flight Mass Spectrometry (MALDI-TOF MS) Based Typing: Employment of Bioinformatics in a Multicenter Study
Source: PLoS One. 2016 Oct 31;11(10):e0164260. doi: 10.1371/journal.pone.0164260 (PMC5087883; doi:10.1371/journal.pone.0164260)

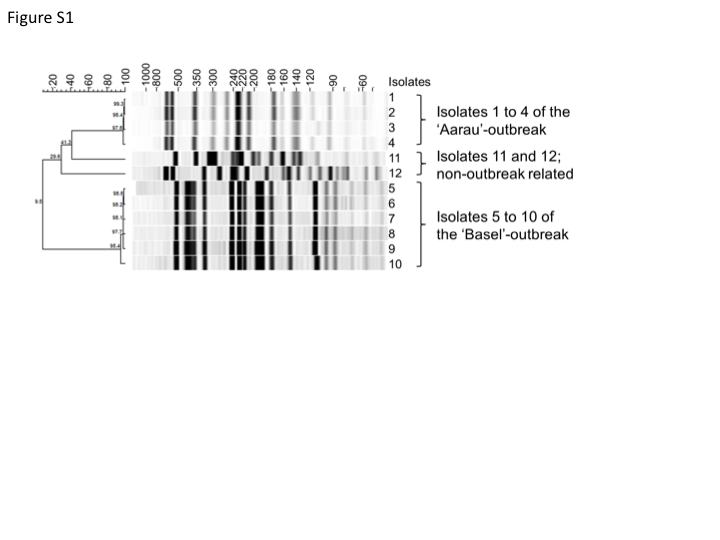

Supplement: S1 Fig — Two outbreak clusters and two non-outbreak related isolates were included into the PFGE analysis. The isolates of the outbreak clusters nicely cluster together (>95% similarity in a pearson’s analysis). The outbreaks were also epidemiologically linked (data not shown). (TIFF) [file pone.0164260.s001.tiff]

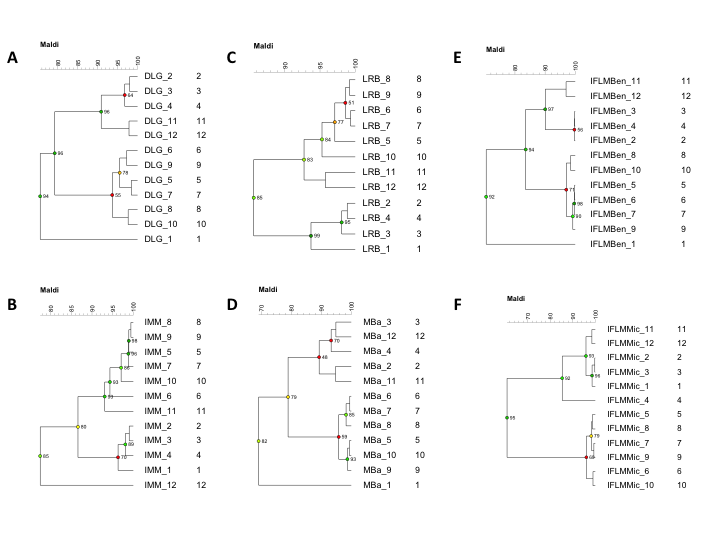

Supplement: S2 Fig — A-G show individuals centers. A, center 1; B, center 2; C, center 3; D, center 4; E, center 5A; F, center 5B. (TIFF) [file pone.0164260.s002.tiff]

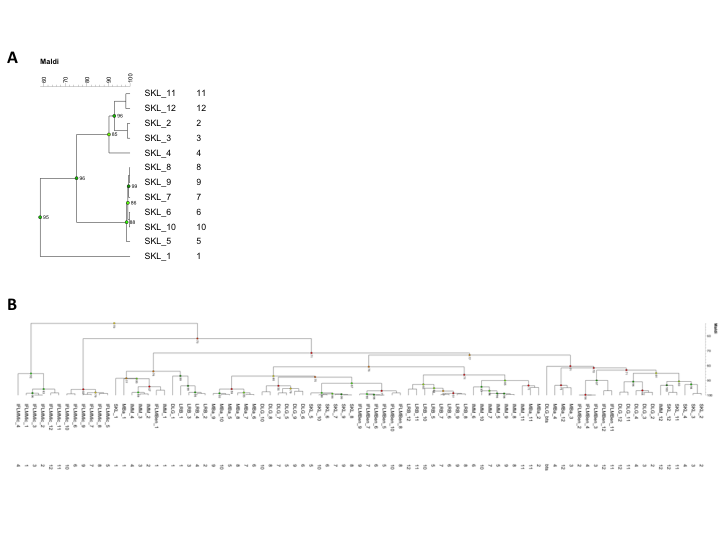

Supplement: S3 Fig — Dendrogram analysis of center 6 and overview A, center 6. B shows the overall dendrogram including all data from all centers. (TIFF) [file pone.0164260.s003.tiff]

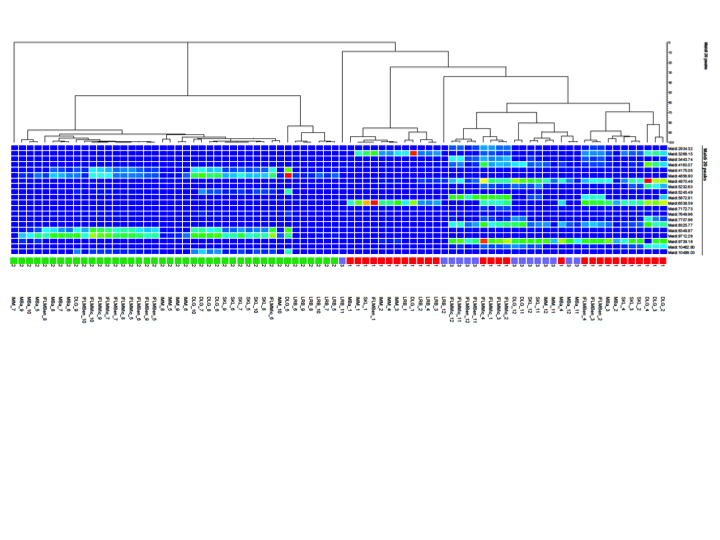

Supplement: S4 Fig — (TIFF) [file pone.0164260.s004.tiff]
