# Supplementary material for: The Technical and Biological Reproducibility of Matrix-Assisted Laser Desorption Ionization-Time of Flight Mass Spectrometry (MALDI-TOF MS) Based Typing: Employment of Bioinformatics in a Multicenter Study
Source: PLoS One. 2016 Oct 31;11(10):e0164260. doi: 10.1371/journal.pone.0164260 (PMC5087883; doi:10.1371/journal.pone.0164260)
Supplement: S1 Table — (DOCX) [file pone.0164260.s005.docx]

**Table S1. Summary of subjective interpretation of discriminant peaks by each center.** Visually identified peaks are shown for each center. The rate of agreement identified the best separating peaks. Rate of agreement gives the percentage of accordance among all centers. The best separating peaks were identified with the peak matching analysis (see Table 2).

| **Peaks** | **1** | **2** | **3** | **4** | **5** | **6** | **7** | **8** | **9** | **10** | **11** | **12** | **13** | **14** | **15** | **16** | **17** | **18** | **19** | **20** | **21** | **22** |
| --- | --- | --- | --- | --- | --- | --- | --- | --- | --- | --- | --- | --- | --- | --- | --- | --- | --- | --- | --- | --- | --- | --- |
| **Center 1** | - | 3092 | - | 5874 | - | - | 6512 | 6539 | - | - | - | - | - | - | 8330 | 8354 | 8372 | 9068 | 9714 | 9741 | 10467 | 10494 |
| **Center 2** | - | - | - | 5872 | - | - | - | 6536 | - | - | 7160 | - | - | - | 8324 | 8350 | - | 9064 | 9710 | 9740 | 10464 | - |
| **Center 3** | - | - | - | - | - | - | - | 6541 | - | - | - | - |  |  | - | 8354 | - | 9070 | 9719 | 9747 | - | - |
| **Center 4** | - | - | - | - | - | 6470 | - | 6540 | 6912 | 7151 | - | 7175 | 7653 | 7710 | 8329 | 8354 | - | - | 9717 | - | 10469 | 10495 |
| **Center 5A** | 3082 | - | 3442 | 5872 | 6436 | - | 6508 | 6537 | - | - | - | 7172 | 7649 | 7707 | 8324 | 8349 | - | - | 9710 | 9736 | 10462 | 10485 |
| **Center 5B** | - | - | - | 5875 | - | - | - | 6540 | - | - | - | - | - | 7708 | 8328 | 8350 | - | - | 9714 | 9742 | 10464 | 10492 |
| **Center 6** | 3082 | - | 3442 | 5873 | 6438 | 6470 | 6509 | 6537 | - | - | - | 7172 | 7651 | 7708 | 8326 | 8351 | - | - | 9714 | 9739 | 10463 | 10491 |
| **Rate of agreement** | 29% | 14% | 29% | 71% | 29% | 29% | 43% | 100% | 14% | 14% | 14% | 43% | 43% | 57% | 86% | 100% | 14% | 43% | 100% | 86% | 86% | 71% |
| **Best separating peaks** |  |  | **3444** | **5873** |  |  |  | **6539** |  |  |  | **7173** | **7650** | **7708** | **8326** | **8350** |  |  | **9712** | **9739** | **10463** | **10489** |
